# Cognitive impairment according to Montreal Cognitive Assessment independently predicts the ability of chronic obstructive pulmonary disease patients to maintain proper inhaler technique

**DOI:** 10.1186/s12890-023-02448-x

**Published:** 2023-04-26

**Authors:** Chonnipha Iamthanaporn, Apiradee Wisitsartkul, Benjamas Chuaychoo

**Affiliations:** 1 Pharmacy Department, Siriraj Hospital, Bangkok, Thailand; 2 Division of Respiratory Disease and Tuberculosis, Department of Medicine, Faculty of Medicine Siriraj Hospital, Mahidol University, 2 Wanglang Road, Bangkoknoi, Bangkok, 10700 Thailand

**Keywords:** Cognitive impairment, Montreal Cognitive Assessment, Incorrect inhaler use, Chronic obstructive pulmonary disease, Pharmacist, Health status

## Abstract

**Background:**

Maintaining correct inhaler technique is crucial in the management of chronic obstructive pulmonary disease (COPD). We aimed to investigate the inhaler technique in patients with COPD, to compare it immediately after and at 1 month after training, and to identify the predictors of incorrect inhaler use at 1 month after training.

**Methods:**

This prospective study was conducted at the COPD clinic of Siriraj Hospital (Bangkok, Thailand). Patients demonstrating improper inhaler use were trained face-to-face by pharmacists. Inhaler technique was re-assessed immediately after and at 1 month after training. The Montreal Cognitive Assessment (MoCA) score, pulmonary function tests, 6-min walk distance (6 MWD), modified Medical Research Council scale score, and COPD Assessment Test (CAT) score were evaluated.

**Results:**

Sixty-six patients with COPD who demonstrated at least one critical error during the use of any controller inhaler were enrolled. The mean age was 73.0 ± 9.0 years, and 75.8% patients had moderate/severe COPD. Immediately after training, all patients used dry powder inhalers correctly and 88.1% used pressurized metered-dose inhalers correctly. At 1 month, the number of patients demonstrating the correct technique decreased across all devices. Multivariable analysis revealed that MoCA score ≤ 16 was independently associated with a critical error at 1 month after training (adjusted odds ratio: 12.7, 95% confidence interval: 1.8–88.2, *p* = 0.010). At 1 month, CAT score (11.4 ± 8.9 *vs*. 8.4 ± 5.5, *p* = 0.018) and 6 MWD (351 ± 93 m *vs*. 372 ± 92 m, *p* = 0.009) had significantly improved in patients demonstrating the correct technique, and CAT score met the minimal clinically important difference.

**Conclusions:**

Face-to-face training by pharmacists improved patient performance. However, the number of patients following proper technique had decreased at 1 month after training. Cognitive impairment (MoCA score ≤ 16) independently predicted the ability of COPD patients to maintain proper inhaler technique. Assessment of cognitive function combined with technical re-assessment and repeated training should improve COPD management.

**Supplementary Information:**

The online version contains supplementary material available at 10.1186/s12890-023-02448-x.

## Introduction

Chronic obstructive pulmonary disease (COPD) is a highly prevalent health condition worldwide. However, pharmacological therapy can reduce the symptoms of COPD, improve exercise tolerance and health status, and prevent exacerbations [1]. Inhalation therapy is the cornerstone of pharmacological treatment, and correct use of inhaler devices is essential. Poor inhalation techniques have been associated with unscheduled use of healthcare resources and poor disease control [2]. Inhalation devices have been widely used for several years. However, errors during the use of inhalers are common [3–5]. Various factors have been reported to be associated with incorrect use of inhalers. These include use of multiple devices, older age, low educational level, reduced manual dexterity and strength, lack of previous inhaler instructions, and cognitive impairment [2, 3, 6–8].

Previous studies have reported that cognitive impairment, which is commonly observed in older adults, is significantly associated with incorrect use of inhalers and reduced ability to retain proper inhalation techniques [3, 6, 7, 9–11]. Reportedly, cognitive impairment (Mini-Mental State Examination [MMSE] score < 24) is associated with incorrect use of inhalers in older adults [6, 10–12]. MMSE and Montreal Cognitive Assessment (MoCA) are commonly used tools for cognitive screening in clinical practice [13, 14]. MMSE is a widely used tool to assess cognitive function among older adults. However, MoCA has a greater sensitivity for detecting mild cognitive impairment (MCI) [14, 15] and more visuoexecutive items [16] compared to MMSE. Patients with COPD are generally older, and these patients were reported to have a 10–77% prevalence of cognitive impairment [17]. However, cognitive screening is not routinely performed for this patient population.

The present study aimed to evaluate the technique of inhaler use in COPD patients immediately after and at 1 month after inhaler use training and to identify the factors that can independently predict incorrect inhaler technique at 1 month after training. Other objectives of this study were to measure the cognitive function of patients included in the study and to evaluate the 1-month short-term effects of correct inhaler use on symptoms of dyspnea, health status, and pulmonary function.

## Materials and methods

### Study design and subjects

This exploratory prospective observational study was conducted at the outpatient COPD clinic of the Division of Respiratory Disease and Tuberculosis, Department of Medicine, Faculty of Medicine, Siriraj Hospital, Mahidol University, Bangkok, Thailand from January 2012 to January 2016. COPD was diagnosed according to the Global Initiative for Chronic Obstructive Lung Disease (GOLD) criteria [1] at 40 years of age. Patients with COPD who regularly used one or more inhalers (pressurized metered-dose inhaler [pMDI], and/or dry powder inhalers [DPI]; Turbuhaler®, Accuhaler®, Handihaler®, and/or Breezhaler®) as maintenance therapy for at least 3 months and those who demonstrated incorrect performance during at least one critical step of the prescribed inhalation technique for at least one controller inhaler were eligible for inclusion. Patients who developed disease exacerbation within 4 weeks before enrollment, those who could not use their inhalers by themselves, those with communication problems, or those using other inhaler devices including pMDI with spacer use were excluded. The study protocol was approved by the Siriraj Institutional Review Board (COA No. Si 014/2012), and written informed consent was obtained from each patient before enrollment.

### Evaluation of inhalation technique

At baseline, enrolled patients were asked to demonstrate the inhalation technique for all the controller inhaler devices prescribed to them. However, placebo versions of each device were used for the demonstrations. Inhalation technique of each patient was assessed by one of the two experienced and trained pharmacists who regularly educate patients regarding the use of inhaler devices at Siriraj Hospital. The pharmacists assessed the inhalation technique using a checklist of critical and non-critical steps during the use of each inhaler type (Supplementary Table 1). Critical errors were defined as those resulting in little or no medication reaching the lungs [18, 19]. The checklist for each inhaler device was modified according to the manufacturer’s instructions and previous studies [18, 19].

Patients who incorrectly performed at least one critical step during the use of any device at baseline were provided with step-by-step inhaler use training by pharmacists for all prescribed inhaler devices. After face-to-face training, patients practiced the inhaler use technique for all of their prescribed inhaler devices until they were able to perform the inhalation technique correctly or up to a maximum of 30 min. After the end of the training, their ability to demonstrate error-free use of all prescribed inhalers was re-assessed. Re-assessment was also performed at 1 month after training. The same pharmacist who evaluated the patients at their first visit evaluated them at the follow-up visit. The results obtained from both the assessments (immediately after training and 1 month after training) were compared to evaluate patient retention of proper inhalation technique.

### Patient data

**Fig. 1:**
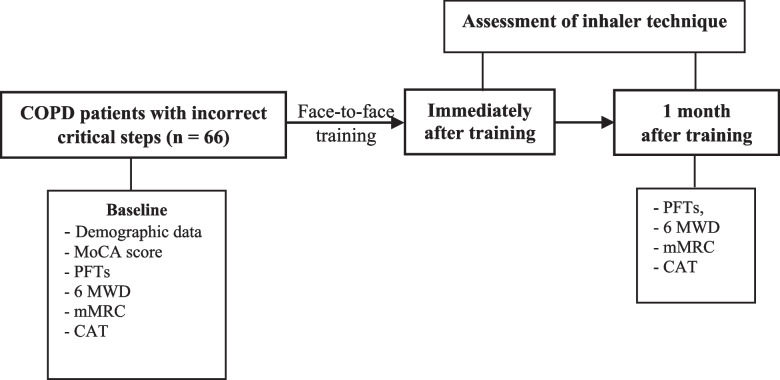
Flow chart of COPD patients with incorrect critical steps (*n* = 66) were trained face-to-face by pharmacists and then assessed the inhalation technique immediately after and at 1 month after training. At baseline, demographic data, MoCA score, PFTs, 6 MWD, mMRC, and CAT of the patients were recorded. At 1 month after training, the patients were evaluated their outcomes (PFTs, 6 MWD, mMRC and CAT)

Data of patients (Fig. 1) who performed at least one incorrect critical step during the use of inhalers were collected and recorded. These included age, sex, educational level, body mass index, smoking status, duration of COPD, severity of COPD, number of inhaler devices used, and duration of use of the prescribed inhalers. History of COPD exacerbations during the 12 months before enrollment was retrieved from the electronic medical record database of the outpatient COPD clinic. COPD exacerbation was defined as acute worsening of respiratory symptoms that resulted in the need for additional therapy. Moderate exacerbation was defined as exacerbation that was treated using short-acting bronchodilators with antibiotics and/or oral corticosteroids. High risk of exacerbation was defined as frequent exacerbations (≥ two moderate exacerbations) and/or severe exacerbations (≥ one hospitalization) within the previous year [1].

At baseline, patients underwent the following pulmonary function tests using the Vmax® Encore system (Viasys Healthcare, Inc.; Yorba Linda, CA, USA): forced expiratory volume in 1 s (FEV_1_), forced vital capacity (FVC), ratio of FEV_1_ and FVC (FEV_1_/FVC), slow vital capacity (SVC), and inspiratory capacity (IC). Severity of dyspnea was assessed using the Thai version of the modified Medical Research Council (mMRC) questionnaire, and health status was evaluated using the Thai version of the COPD Assessment Test (CAT) [20] and 6-min walk distance (6 MWD). An mMRC scale score ≥ 2 and a CAT score ≥ 10 are considered to indicate profound symptoms according to the GOLD guidelines [1]. The Thai version of MoCA [21] was used to assess cognitive impairment at baseline. The cognitive domains of MoCA include visuospatial/executive functions, naming, memory, attention, language, abstraction, delayed recall, and orientation. The MoCA scores ranged from 0 to 30, and scoring was performed by an investigator trained and certified to administer the MoCA questionnaire. Any questionnaire under copyright protection was administered after permission was granted by the copyright holder.

At 1 month after training, patients’ ability to correctly perform the steps of the inhalation technique for each of their prescribed inhalers was re-assessed by the same pharmacist who performed the assessment immediately after training. Potential factors associated with incorrect critical steps at 1 month after training were analyzed. These included age, educational level, duration of COPD, severity of COPD (FEV_1_% predicted), mMRC score, CAT score, frequent and/or severe exacerbations within the previous year, number of inhaler devices, duration of use of the prescribed inhalers, and MoCA score at baseline. We also evaluated short-term outcomes in patients who correctly used at least one inhaler containing a bronchodilator (controller device) at 1 month after training. Results of pulmonary function tests, 6 MWD, mMRC scale scores, and CAT scores were collected at baseline and at 1 month after training by technicians who were blinded to the results of training (Fig. 1).

### Sample size calculation and statistical analysis

Only the patients with COPD who demonstrated at least one incorrect critical step during the use of any controller inhaler were enrolled. They were provided training regarding the use of inhalers, and their technique was evaluated immediately after and at 1 month after training. We estimated the sample size by using a 95% level of confidence. Altogether, 50% of the patients demonstrated correct inhalation technique after intervention (expected prevalence), 15% performed it with relative precision, and a potential loss of follow up 15% was recorded. The estimated sample size was 50 patients. Baseline patient characteristics were summarized using descriptive statistics. Categorical data were compared using the chi-squared test or Fisher’s exact test, and the results were presented as numbers and percentages. Continuous data with normal distribution were compared within group and between two groups by using a paired *t*-test and a student sample *t*-test, respectively, and the outcomes were presented as mean ± standard deviation. McNemar’s test was used to compare the proportional outcomes immediately after and at 1 month after training.

Receiver operating characteristic (ROC) curve analysis with area under the curve and Youden’s index was performed to obtain the optimal cut-off value of the MoCA score for predicting incorrect inhalation technique at 1 month after training. Covariates with a *p*-value < 0.25 [22] in the univariable analysis were included in the multivariable analysis using a multivariable binary logistic regression model to identify the factors independently associated with incorrect inhalation technique at 1 month after training. The results of these analyses were reported as odds ratios (ORs) and 95% confidence intervals (CIs) for the univariable analysis and as adjusted odds ratios (aORs) and 95% CIs for the multivariable analysis. A *p*-value < 0.05 was considered statistically significant. All statistical analyses were performed using PASW Statistics 18.0 (SPSS Inc., Chicago, IL, USA).

## Results

**Fig. 2:**
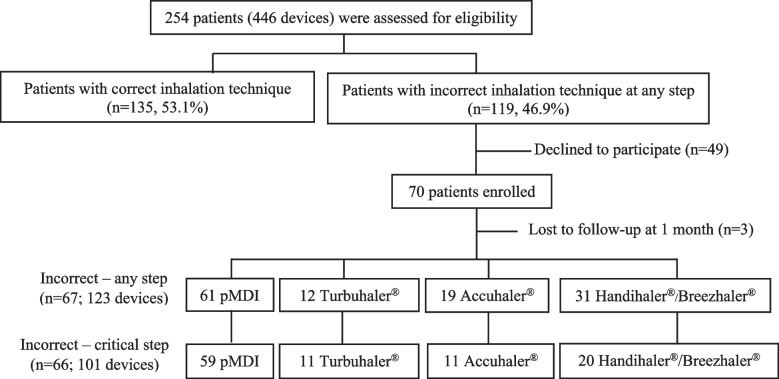
Flowchart of chronic obstructive pulmonary disease patients who incorrectly performed any critical step of an inhaler use technique for any study inhaler. A total of 66 patients who failed to perform a critical step were enrolled in this study

Altogether, 254 patients with COPD using 446 inhaler devices (197 pMDIs, 93 Handihalers®, 89 Accuhalers®, 38 Turbuhalers®, and 29 Breezhalers®) were assessed for the performance of their inhalation technique at the outpatient COPD clinic of our center. Incorrect inhalation technique at any step during the use of any inhaler was observed in 119 out of 254 (46.9%) patients (Fig. 2). Among the 446 inhaler devices, most of the errors occurred during the use of pMDIs (104 out of 197, 52.8%), followed by Turbuhalers® (16 out of 38, 42.1%), Handihalers® (30 out of 93, 32.3%), Accuhalers® (26 out of 89, 29.2%), and Breezhalers® (7 out of 29, 24.1%).

Seventy patients who incorrectly performed any step during the use of an inhaler consented to participate in the study. Three patients were lost to follow-up at 1 month after training. Among the remaining 67 patients, 66 (101 devices) incorrectly performed at least one critical step while using any inhaler, and these patients were included in the final analysis. The 101 devices comprised of 59 pMDIs, 11 Turbuhalers®, 11 Accuhalers®, and 20 Handihalers®/Breezhalers® (Fig. 2). We combined the 15 Handihaler® and five Breezhaler® devices in the same group for the analysis, since the inhalation techniques for these two devices were similar. Most of the patients (62 out of 66, 93.9%) had received inhalation instructions or training from physicians or pharmacists before enrollment.

### Baseline characteristics

The mean age of the 66 patients included in the final analysis was 73.0 ± 9.0 years (range: 51–93 years), and 52 (78.8%) were aged ≥ 65 years (Table 1). Sixty-three patients (95.5%) were male and 36 (54.5%) had a low educational level (≤ 6 years). Thirty-eight (57.6%) patients were diagnosed with COPD for > 3 years, 50 patients (75.8%) had moderate to severe COPD, and 20 patients (30.3%) had frequent exacerbations and/or hospitalization within the previous year. Most of the patients (47, 71.2%) were prescribed ≥ 2 different inhaler devices. A combination of one pMDI and DPI was the most common inhaler combination (36, 54.5%). Most of the patients (55, 83.3%) had used the prescribed inhalers for > 6 months. The mean MoCA score was 19.3 ± 4.8 among patients who demonstrated critical errors at baseline. According to the study by Hemrungrojn et al. [23], MCI (MoCA score ≤ 24) was observed in 46 out of 57 (80.7%) patients. A MoCA score ≤ 16 (considered to indicate the presence of dementia) [23] was observed in 16 out of 57 (28.1%) patients. The most commonly impaired MoCA domain was delayed recall (1.4 ± 1.6 out of 5 points, percentage score: 28.4), followed by language (1.0 ± 1.1 out of 3 points, percentage score: 32.7), and visuospatial/executive functions (2.8 ± 1.4 out of 5 points, percentage score: 55.8) (Table 1).

**Table 1:** Baseline characteristics of COPD patients who incorrectly performed a critical step of a prescribed inhalation technique for any one inhaler device before receiving inhalation training by a pharmacist (*N* = 66)

Characteristics	*n* = 66	Characteristics	*n* = 66
**Age** (years), (min, max)	73 ± 9 (51, 93)	**Number of device models**	
Age (years)		1 device	19 (28.8)
≥ 65	52 (78.8)	2 devices	38 (57.6)
< 65	14 (21.2)	3 devices	9 (13.6)
**Sex**: Male	63 (95.5)	**Type of devices**	
**BMI** (kg/m^2^)	21.6 ± 3.9	pMDI only	16 (24.2)
**Education level**		pMDI + 1 DPI	36 (54.5)
Lower education (≤ 6 years)	36 (54.5)	pMDI + 2 DPIs	9 (13.6)
Higher education (> 6 years)	30 (45.5)	DPIs only	3 (4.5)
**Smoking status**		2 DPIs	2 (3.0)
Never smokers	1 (1.5)	**Duration of prescribed inhalers**	
Ex-smokers	57 (86.4)	≤ 6 months	11 (16.7)
Current smokers	8 (12.1)	> 6 months	55 (83.3)
**COPD duration** (years)		**Mean MoCA score** (*n* = 57)	19.3 ± 4.8 (10, 28)
< 1	8 (12.1)	**MoCA score**^c^(*n* = 57)	
1—3	20 (30.3)	≤ 24 points	46 (80.7)
> 3	38 (57.6)	> 24 points	11 (19.3)
**FEV**_**1**_**/FVC** (%)	50.6 ± 11.3	≤ 16 points	16 (28.1)
**Severity of COPD**^a^		> 16 points	41 (71.9)
Mild	14 (21.2)	**MoCA domain score**, mean ± SD, (percentage score)^d^	
Moderate	32 (48.5)	Visuospatial/executive (5 points)	2.8 ± 1.4 (55.8)
Severe	18 (27.3)	Naming (3 points)	2.8 ± 0.5 (94.7)
Very severe	2 (3.0)	Attention (6 points)	4.2 ± 1.5 (70.2)
**mMRC**, median (IQR)	1.0 (0–2)	Language (3 points)	1.0 ± 1.1 (32.7)
mean ± SD (min,max)	1.2 ± 1.3 (0, 4)	Abstraction (2 points)	1.2 ± 0.8 (58.8)
**CAT**, (min,max)	11.3 ± 8.0 (1, 33)	Delayed recall (5 points)	1.4 ± 1.6 (28.4)
**Exacerbations** in 1 year^b^	20 (30.3)	Orientation (6 points)	5.4 ± 0.9 (89.8)
**Previous inhaler education**	62 (93.9)		

Data presented as number (percentage) or mean ± standard deviation (min, max) unless otherwise indicated

^a^GOLD classification of COPD severity by post-bronchodilator FEV1% predicted, Mild (≥ 80), Moderate (50–79), Severe (30–49), Very Severe (< 30) [1]

^b^Frequent exacerbations (≥ 2 moderate exacerbations) and/or ≥ 1 hospitalization in 1 year prior enrollment

^c^MoCA score ≤ 24 points and ≤ 16 points are considered mild cognitive impairment (MCI) and dementia, respectively [23]

^d^Percentage score = a mean percentage score calculated as a percentage of maximum possible score

### Critical errors at baseline and at 1 month after training

At baseline, the most common critical errors among the users of pMDIs were during the following steps: “inhale slowly and deeply” (50 out of 59, 84.7%) and “press the canister once and inhale at the same time” (40 out of 59, 67.8%) (Table 2). The most common critical errors among the users of Turbuhaler® were during the following steps: “keep the inhaler in an upright position” (8 out of 11, 72.7%) and “inhale forcefully and deeply” (7 out of 11, 63.6%). The most common critical errors among the users of Accuhaler® were during the following step: “breathe out gently away from the inhaler to residual volume” (9 out of 11, 81.8%). The most common critical errors among the users of Handihaler®/Breezhaler® were during the following step: “pierce the capsule by fully pressing the button once and then release” (19 out of 20, 95.0%). At 1 month after training, critical errors were significantly decreased across all devices compared to baseline (Table 2).

**Table 2:** The number of COPD patients who incorrectly performed a critical step of a prescribed inhalation technique compared between before inhaler use training (baseline) and 1 month after inhaler use training (*N* = 66)

**Critical steps**^a^	**Incorrect Technique**			
	**Before training, n (%)**		**1 month after training, n (%)**	***P-*value**
**pMDI (*n* = 59)**				
1. Remove cap	0 (0)		0 (0)	N/A
2. Keep inhaler in upright position	3 (5.1)		0 (0)	0.250
3. Shake inhaler vertically 4–5 times	24 (40.7)		9 (15.3)	0.001*
4. Press the canister 1 time and inhale at the same time	40 (67.8)		24 (40.7)	< 0.001*
5. Inhale slowly and deeply	50 (84.7)		35 (59.3)	0.001*
*** 6. Error at any step***	***59 (100.0)***		***49 (83.1)***	0.002*
**Turbuhaler (*n* = 11)**				
1. Remove cap	0 (0)		0 (0)	N/A
2. Keep inhaler in upright position	8 (72.7)		5 (45.5)	0.250
3.Turn red grip anti-clockwise then turn back until it “click”	5 (45.5)		0 (0)	0.063
4. Breath out gently away from the inhaler to residual volume	4 (36.4)		1 (9.1)	0.250
5. Inhale forcefully and deeply	7 (63.6)		3 (27.3)	0.125
*** 6. Error at any step***	***11 (100.0)***		***5 (45.5)***	0.031*
**Accuhaler (*n* = 11)**				
1. Open cover	0 (0)		0 (0)	N/A
2. Push the lever until it “click”	3(27.3)		1 (9.1)	0.500
3. Breath out gently away from the inhaler to residual volume	9 (81.8)		1 (9.1)	0.008*
4. Inhale forcefully and deeply	3 (27.3)		1 (9.1)	0.500
*** 5. Error at any step***	***11 (100.0)***		***3 (27.3)***	0.008*
**Handihaler/Breezhaler (*n* = 20)**				
1. Open cover	0 (0)		0 (0)	N/A
2. Insert the capsule into the chamber	0 (0)		0 (0)	N/A
3. Pierce the capsule by fully pressing the button 1 time and then release		19 (95.0)	8 (40.0)	0.001*
4. Breath out gently away from the inhaler to residual volume	5 (25.0)		1 (5.0)	0.219
5. Inhale deeply with capsule vibrating sound	6 (30.0)		3 (15.0)	0.453
*** 6. Error at any step***	***20 (100.0)***		***9 (45.0)***	0.001*

*N/A* Not applicable

^*^ *P*-value < 0.05 by McNemar's test

^a^Modified from previous study [18, 19]

### Correct inhaler technique immediately after and at 1 month after training

**Fig. 3:**
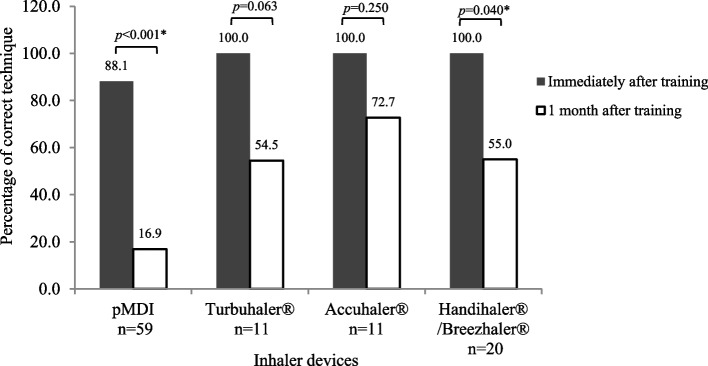
Percentage of patients who correctly performed all critical steps compared between immediately after and 1 month after inhaler use training for each type of inhaler device (total *N* = 66)

The face-to-face training included up to 30 min of practice time. Immediately after the end of training, the pharmacists re-assessed the inhalation technique of each patient for his/her prescribed inhalers. All patients using DPIs (Turbuhaler®, Accuhaler®, and Handihaler®/Breezhaler®) correctly performed all the critical steps immediately after training. In contrast, only 52 out of 59 (88.1%) patients using pMDIs performed the inhalation technique correctly immediately after training (Fig. 3). At 1 month after training, the percentage of patients who correctly performed all the critical steps had decreased across all devices and significantly decreased in case of pMDIs (52 [88.1%] *vs*. 10 [16.9%], *p* < 0.001) and Handihalers®/Breezhalers® (20 [100.0%] *vs*. 11 [55.0%], *p* = 0.040). Accuhaler® was associated with the lowest decrease in the number of patients who could correctly perform all the critical steps at 1 month after training when compared with the results immediately after training (11 [100.0%] *vs*. 8 [72.7%], *p* = 0.250].

### Factors associated with critical errors at 1 month after training

Potential factors associated with critical errors at 1 month after training were evaluated. MoCA was not used in 9 out of 66 patients at baseline (4 patients in the correct technique group and 5 patients in the incorrect technique group at 1 month after training). The total MoCA score in patients using incorrect techniques was significantly lower than that in patients using the correct technique at 1 month after training (18.1 ± 5.3 *vs*. 20.7 ± 3.8, *p* = 0.034) (Table 3). The MoCA score of each domain in patients using incorrect techniques tended to be lower than that in patients using the correct technique, but the difference was statistically significant only for the attention domain (3.7 ± 1.6 *vs*. 4.7 ± 1.2, *p* = 0.011). We evaluated the cut-off value of the MoCA score using ROC analysis and observed that MoCA score > 16 was the optimal cut-off for discriminating between patients with and without incorrect inhalation techniques at 1 month after training. The area under the ROC curve was 0.643 (95% CI: 0.497–0.788), with 92.6% sensitivity and 46.7% specificity (supplementary Fig. 1)*.* Youden’s index of > 16 points was the maximum (0.393). We also analyzed both the cut-off points for the MoCA score (≤ 24 defined as MCI and ≤ 16 defined as dementia) [23].

**Table 3:** Comparison of scores from different cognitive domains of Montreal Cognitive Assessment between patients with incorrect technique and those with correct technique at 1 month after inhaler use training

**MoCA domains**		**Incorrect technique** **at 1 month after training (*n* = 30)**		**Correct technique** **at 1 month after training (*n* = 27)**		***p*-value**
		**Mean ± SD**	**Percentage score**^a^	**Mean ± SD**	**Percentage score**^a^	
Delayed recall	(5 points)	1.2 ± 1.5	24.0	1.7 ± 1.6	33.3	0.259
Language	(3 points)	0.8 ± 1.1	27.8	1.1 ± 1.1	38.3	0.289
Visuospatial/executive	(5 points)	2.5 ± 1.5	49.3	3.1 ± 1.2	63.0	0.068
Abstraction	(2 points)	1.1 ± 0.8	53.3	1.3 ± 0.7	64.8	0.258
Attention	(6 points)	3.7 ± 1.6	62.2	4.7 ± 1.2	79.0	0.011*
Orientation	(6 points)	5.3 ± 1.0	88.3	5.5 ± 0.8	91.4	0.472
Naming	(3 points)	2.8 ± 0.5	94.4	2.9 ± 0.5	95.1	0.880
**Total score**	**(30 points)**	**18.1** ± **5.3**	**60.2**	**20.7** ± **3.8**	**69.0**	0.034*

^a^The percentage score was calculated using the mean score/maximum possible score in each cognitive domain

^*^ *p* < 0.05, suggesting statistical significance

Univariable analysis showed that age > 75 years (OR: 3.8, 95% CI: 1.3–10.9, *p* = 0.010) and MoCA score ≤ 16 (OR: 10.9, 95% CI: 2.2–54.7, *p* = 0.001) were significantly associated with an incorrect technique of using any controller inhaler device (at least one critical error) at 1 month after training (Table 4). Multivariable logistic regression analysis revealed that MoCA score ≤ 16 was the only independent predictor of at least one critical error during the use of any inhaler (aOR: 12.7, 95% CI: 1.8–88.2, *p* = 0.010) after adjustment for age, educational level, duration of COPD, mMRC scale score, and duration of use of the prescribed inhaler (Table 4).

**Table 4:** Univariable and multivariable analysis to determine significant risk factors for incorrect performance at any critical step of a prescribed inhalation technique at 1 month after inhaler use training

Univariable (total *n* = 66)					Multivariable (*n* = 57)	
**Variables**	**n**	**Incorrect, n (%)**	**OR (95% CI)**	***p*-value**	**aOR (95% CI)**	***p*-value**
**Age**						
> 75 years	28	20 (71.4)	3.8 (1.3–10.9)	0.010*	1.9 (0.5–7.3)	0.345
≤ 75 years (Ref)	38	15 (39.5)				
**Education**						
Low (≤ 6 years)	36	22 (61.1)	2.1 (0.8–5.5)	0.150	1.2 (0.3–4.5)	0.760
High (> 6 years) (Ref)	30	13 (43.3)				
**Duration of disease**						
> 3 years	38	23 (60.5)	2.0 (0.8–5.5)	0.155	3.5 (0.8–16.2)	0.105
≤ 3 years (Ref)	28	12 (42.9)				
**Baseline FEV**_**1**_						
< 50% predicted	20	12 (60.0)	1.5 (0.5–4.4)	0.454		
≥ 50% predicted (Ref)	46	23 (50.0)				
**Baseline mMRC scale score**						
≥ 2	27	17 (63.0)	2.0 (0.7–5.4)	0.179	1.1 (0.3–4.9)	0.877
< 2 (Ref)	39	18 (46.1)				
**Baseline CAT score**						
≥ 10	26	15 (57.7)	1.4 (0.5–3.7)	0.541		
< 10 (Ref)	40	20 (50.0)				
**Frequent exacerbations**						
Yes	20	10 (50.0)	0.8 (0.3–2.4)	0.745		
No (Ref)	46	25 (54.3)				
**Number of devices**						
≥ 2 devices	47	23 (48.9)	0.6 (0.2–1.7)	0.295		
< 2 devices (Ref)	19	12 (63.2)				
**Prescribed inhalers**						
Newly prescribed (≤ 6 months)	11	3 (27.3)	0.3 (0.1–1.1)	0.061	0.5 (0.1–3.5)	0.479
Long standing (> 6 months) (Ref)	55	32 (58.2)				
**MoCA score**						
≤ 24	46	25 (54.3)	1.4 (0.4–5.4)	0.596		
> 24 (Ref)	11	5 (45.5)				
≤ 16	16	14 (87.5)	10.9 (2.2–54.7)	0.001*	12.7 (1.8–88.2)	0.010*
> 16 (Ref)	41	16 (39.0)				

### Short-term clinical outcomes in patients with correct inhaler use technique

The inhalation technique, results of pulmonary function tests (FVC, FEV_1_, FEV_1_/FVC, SVC, and IC), exercise capacity (6 MWD), dyspnea score (mMRC scale score), and health status (CAT score) were re-evaluated at 1 month after training (Fig. 1). We compared these parameters between patients who correctly performed (31, 47%) the prescribed inhalation technique for at least one device containing a bronchodilator and patients who incorrectly performed (35, 53%) for all prescribed devices containing bronchodilators at 1 month after training (Table 5). Significant improvement was observed in the CAT score (11.4 ± 8.9 *vs*. 8.4 ± 5.5, *p* = 0.018) and 6 MWD (351 ± 93 m *vs*. 372 ± 92 m, *p* = 0.009) at 1 month when compared with the baseline values in patients who correctly performed the prescribed technique at 1 month after training (Table 5). Dyspnea (mMRC score) did not improve significantly. Most of the parameters from pulmonary function tests did not change significantly. However, IC was significantly increased (1.6 ± 0.5 *vs*. 1.7 ± 0.5, *p* = 0.014) in patients with incorrect technique.

**Table 5:** Comparison of baseline (before training) pulmonary function test parameters, 6-min walk distance, modified Medical Research Council scale scores, and Chronic obstructive pulmonary disease Assessment Test scores with those at 1 month after inhaler use training in the correct and incorrect technique groups^a^

Correct group (*n* = 31)				Incorrect group (*n* = 35)			
**Variables**	**Baseline**	**1 month**	***p*-value**	**Variables**	**Baseline**	**1 month**	***p*-value**
**PFTs** (*n* = 27)^b^				**PFTs** (*n* = 31)^b^			
FEV_1_ (L)	1.3 ± 0.4	1.4 ± 0.4	0.147	FEV_1_ (L)	1.2 ± 0.4	1.3 ± 0.4	0.380
FVC (L)	2.6 ± 0.5	2.7 ± 0.5	0.194	FVC (L)	2.4 ± 0.5	2.5 ± 0.5	0.226
FEV_1_/FVC (%)	50.5 ± 12.1	50.9 ± 12.3	0.547	FEV_1_/FVC (%)	50.7 ± 11.4	50.4 ± 11.5	0.652
SVC (L)	2.7 ± 0.5	2.7 ± 0.5	0.317	SVC (L) (*n* = 30)	2.4 ± 0.5	2.5 ± 0.5	0.242
IC (L)	1.8 ± 0.4	1.8 ± 0.4	0.474	IC (L) (*n* = 30)	1.6 ± 0.5	1.7 ± 0.5	0.014*
**mMRC scale score**	1.2 ± 1.3	1.4 ± 1.3	0.395	**mMRC scale score**	1.3 ± 1.2	1.4 ± 1.2	0.711
**CAT score**	11.4 ± 8.9	8.4 ± 5.5	0.018*	**CAT score**	11.1 ± 7.3	10.7 ± 7.4	0.655
**6 MWD**^b^ (*n* = 26)	351 ± 93	372 ± 92	0.009*	**6 MWD**^b^ (*n *= 30)	310 ± 103	310 ± 103	0.998

Data are presented as mean ± standard deviation unless otherwise indicated

^*^p < 0.05 according to paired t-test

^a^The correct group included patients who correctly performed all the critical steps for at least one device containing a bronchodilator. The incorrect group included patients who incorrectly performed the techniques for all prescribed devices containing bronchodilators at 1 month after training

^b^Four patients in each group did not consent to undergo PFTs, and 5 patients in each group did not consent to the measurement of 6 MWD

## Discussion

Almost 50% of the patients with COPD in our clinic performed the inhalation technique for their inhalers incorrectly, although most of them (93.9%) had received prior inhalation instruction or training. However, the percentage of patients with an incorrect technique was significantly decreased across all devices at 1 month after receiving face-to-face training regarding the use of inhalers (Table 2). This finding is consistent with those from previous studies suggesting that repeated training in inhalation techniques can improve inhalation performance [3, 24, 25].

The pMDI was the most frequently used type of inhaler in our outpatient COPD clinic. However, we found that pMDIs were associated with the highest rate of incorrect use, which is consistent with the findings reported in previous studies [25–28]. Previous study [25] showed that the use of a spacer with pMDI can reduce the error of inhalation technique in COPD patients (69.7% of 33 patients using pMDI with a spacer *vs.* 77.3% of 44 patients using pMDI without a spacer). However, we did not include the patients using pMDI with spacer in our study. We conducted the study based on inhaler devices are regularly used by patients in a real-world practice of our COPD clinic, which a relatively low percentage of patients (12.3%, 37 of 300 patients using pMDI) regularly use a spacer.

The most common error during the use of pMDIs (84.7%) was during the “inhale slowly and deeply” step. Patients should inhale slowly and deeply for 3–5 s to reduce the impact of medication on the oropharynx and hypopharynx. This type of inhalation is equivalent to inhalation with SVC [29]. This technique might be used in cases where proper inspiratory flow detection measures (such as the In-Check DIAL) are not available.

Immediate assessment after face-to-face training for up to 30 min revealed that only 88.1% of the patients were able to use the pMDIs correctly, while all patients were able to use the DPIs (Turbuhaler®, Accuhaler®, Handihaler®, and Breezhaler®) correctly. At 1 month after training, the percentage of patients demonstrating the correct technique decreased across all devices. The pMDIs had the lowest rate of retention of correct inhalation technique, while Accuhaler® had the highest rate of retention (Fig. 3). These findings suggest that re-assessment of inhalation techniques, repeated training, and selection of suitable and individualized inhalation devices are necessary [30, 31].

### Factors associated with incorrect inhaler technique at 1 month after training

According to the univariable analysis, age > 75 years and MoCA score ≤ 16 were significantly associated with incorrect inhaler technique at 1 month after training. However, multivariable analysis revealed that only MoCA score ≤ 16 was independently associated with incorrect inhalation technique (aOR: 12.7, 95% CI: 1.8–88.2, *p* = 0.010) after adjustment for age, educational level, duration of COPD, mMRC scale score, and duration of use of the prescribed inhaler (Table 4). The optimal MoCA score cut-off point of 16 in this study was derived from the optimal area under the ROC curve (0.643, 95% CI: 0.497–0.788) and maximum Youden’s index of 0.393. This cut-off point was the same as that reported by Hemrungrojn et al. to define dementia (≤ 16) [23]. Nevertheless, the wide confidence interval (95% CI: 1.8–88.2) for MoCA score ≤ 16 might suggest a small sample size for the multivariable analysis.

Previous studies have reported that older age, low educational level, higher mMRC scale score, lower CAT score, and greater severity of COPD (FEV_1_% predicted value) were associated with poor inhalation technique [25, 31, 32]. However, none of these factors showed statistical significance in our study. This might be because we studied a specific group of patients with COPD and only those demonstrating incorrect inhaler techniques were trained and re-assessed at 1 month after training. We observed that cognitive impairment (measured using the MoCA score) was the only independent predictor of incorrect inhalation techniques in older patients with COPD. This is consistent with the finding reported by Maricoto et al. [28] who reported that cognitive impairment assessed using the MoCA score was one of the factors that influenced the accuracy of an inhalation technique in older adults with asthma or COPD.

Previous studies have reported that cognitive impairment (MMSE score < 24) is associated with incorrect inhalation techniques in older patients [6, 10–12], older patients with COPD and asthma [31], and patients with COPD [33]. Both MMSE and MoCA are cognitive screening tests. However, we used MoCA to detect cognitive impairment including MCI [14, 15, 34]. Recently, Hemrungrojn et al. [23] reported that the best cut-off score of the Thai version of MoCA was ≤ 24 for discriminating amnestic MCI from healthy controls and > 16 from Alzheimer’s disease. We observed that MoCA scores ≤ 24 were not significantly associated with an incorrect inhalation technique at 1 month after training. Taken together, these findings suggest that some patients with MCI can be trained to use an inhaler correctly. However, patients with MoCA scores ≤ 16 might not be able to use inhaler devices correctly by themselves. Luley et al. [35] showed that an 8-day intervention comprising of daily counseling and video demonstration in patients having COPD with MCI could improve the inhaler technique, while patients with severe cognitive deficits were unable to reduce the number of mistakes during the use of inhalers. These findings suggest that repeat training may be beneficial for patients with MCI. However, in patients with dementia who are unable to perform the inhalation technique accurately, alternative treatment options for drug administration such as using a nebulizer should be considered [30, 36]. Additionally, some patients may require caregivers to perform all the steps before the patients inhale the medication.

### Cognitive impairment according to Montreal Cognitive Assessment in patients with chronic obstructive pulmonary disease who showed incorrect inhaler use

We found that the incidence of cognitive impairment (MoCA score ≤ 16, suggesting dementia) [23] was 28.1% (Table 1), which was higher than the prevalence of dementia among elderly Thai individuals in various population-based studies in Thailand (range: 3.3–9.9%) [37–39]. However, Liao et al. [40] showed that patients with COPD had an increased risk of dementia compared to those without COPD after adjusting for age, sex, and comorbidities (adjusted hazard ratio: 1.74, 95% CI: 1.55–1.96). The high rate of dementia in our study might be due to inclusion of a specific group of patients with COPD and also because only the patients showing incorrect inhalation techniques were enrolled.

The MoCA domains showing the highest impairment in our study were delayed recall, language, and visuospatial/executive functions (Table 1). Patients with impairment in memory and executive functions might have difficulties in remembering how to use the inhaler and execute the correct technique [6, 10, 41]. Furthermore, total MoCA scores and scores in the attention domain were significantly lower in patients with persistent incorrect performance than in those showing the correct technique at 1 month after training (Table 3). Deficits in attention and frontal-executive functions may result in impaired self-management [41].

The prescribed technique for proper use of inhalers, especially for pMDIs in this study, may have been too complex for patients with cognitive impairment, since they were unable to learn and retain the correct steps of the inhalation protocol [6, 9–11, 33]. Simpler steps of inhaler use may improve the technique in patients with cognitive impairment. Patients with incorrect inhalation techniques should be assessed for cognitive function. Moreover, they should have their inhalation technique re-assessed regularly and undergo repeated inhaler use training [30, 31].

### Short-term clinical outcomes in patients with correct inhalation technique

Since bronchodilators are the mainstay of pharmacological treatment for COPD to relieve symptoms and improve exercise tolerance and health status [42], patients who correctly performed the prescribed technique of at least one device containing a bronchodilator might benefit from the treatment. We found that symptoms of dyspnea (mMRC scale score) did not change significantly. However, 6 MWD and CAT scores improved significantly from baseline to 1 month after training in patients with COPD who showed the correct technique (Table 5). However, only health status (CAT score) met the minimal clinically important difference (MCID, a decrease of 2 points) [43] (11.4 ± 8.9 *vs*. 8.4 ± 5.5, *p* = 0.018). The pulmonary function test parameters except IC did not change significantly. IC in patients with incorrect technique improved significantly (1.6 ± 0.5 L *vs*. 1.7 ± 0.5 L,* p* = 0.014), but it did not meet the MCID (an increase of 200 ml) [44]. Previous studies regarding the effect of inhaler training on dyspnea, health status, and quality of life varied in terms of assessment duration, and the results of these studies were often conflicting. Some studies did not find any improvement [24, 45], while others did [35, 46].

### Limitations

The present study has some limitations. We did not perform direct measurement of the inspiratory flow (such as measurement using the In-Check DIAL) to evaluate the optimal inspiratory flow for each device. The inhalation technique for the pMDIs was evaluated by experienced pharmacists who instructed the patients inhale slowly and deeply for 3–5 s. The techniques for using Accuhaler® and Turbuhaler® were evaluated using device trainer whistles provided by the manufacturers. The techniques for using Handihaler® and Breezhaler® were evaluated by hearing the sound of the capsule vibrating in the chamber during inhalation and checking for the absence of powder in the capsule after the inhalation maneuver. Most of the inhalers prescribed in our clinic are pMDIs. Hence, the sample size for other devices may have been smaller than that required to yield statistically reliable results. We assessed the 1-month short-term clinical outcomes. This follow-up duration might be too short to evaluate all clinical outcomes of the treatment. The wide confidence interval (95% CI: 1.8–88.2) for the MoCA score ≤ 16 in the multivariable analysis might suggest a small sample size. Thus, a larger sample size is required in future studies.

## Conclusions

Face-to-face inhaler use training by pharmacists improves patient performance. However, the percentage of patients using the proper technique had decreased significantly at 1 month after training in the present study. We observed that cognitive impairment (MoCA score ≤ 16) could independently predict incorrect use of inhalers. Assessment of cognitive function, device selection, re-assessment of the technique, and repeated training should improve COPD management. Further studies are needed to investigate the appropriate inhaler devices and techniques for COPD patients with cognitive impairment.

## Supplementary Information

**Additional file 1: Supplementary Table 1.** Inhaler use checklists for the 4 groups of study inhalers.

**Additional file 2: Supplementary Fig. 1.** Receiver operating characteristic curve analysis of the Montreal Cognitive Assessment scores in patients with and without incorrect technique at 1 month after training. The optimal cut-off value was 16. 

## Data Availability

The datasets analyzed during the current study are available from the corresponding author upon reasonable request.

## Abbreviations

aOR: Adjusted odds ratio
AUC: Area under the curve
BMI: Body mass index
CAT: COPD assessment test
CI: Confidence interval
COPD: Chronic obstructive pulmonary disease
DPI: Dry powder inhalers
FEV_1_: Forced expiratory volume in 1 s
FVC: Forced vital capacity
IC: Inspiratory capacity
GOLD: Global initiative for chronic obstructive lung disease
MCI: Mild cognitive impairment
MMSE: Mini-mental state examination
mMRC: Modified Medical Research Council dyspnea scale
MoCA: Montreal cognitive assessment
OR: Odds ratio
pMDI: Pressurized metered-dose inhaler
PFTs: Pulmonary function tests
ROC: Receiver operating characteristic
SD: Standard deviation
SVC: Slow vital capacity
6 MWD: 6-Minute walk distance
