# Supplementary material for: Cognitive impairment according to Montreal Cognitive Assessment independently predicts the ability of chronic obstructive pulmonary disease patients to maintain proper inhaler technique
Source: BMC Pulm Med. 2023 Apr 26;23:144. doi: 10.1186/s12890-023-02448-x (PMC10131352; doi:10.1186/s12890-023-02448-x)
Supplement: Supplementary file 1 — Additional file 1: Supplementary Table 1. Inhaler use checklists for the 4 groups of study inhalers. [file 12890_2023_2448_MOESM1_ESM.pdf]

Supplementary Table 1. Inhaler use checklists for the 4 groups of study inhalers

pMDI

1. **Remove cap**
2. **Keep inhaler in upright position**
3. **Shake inhaler vertically 4-5 times**
4. Place the mouthpiece between the lips and teeth to seal the mouthpiece
5. Breath out gently to residual volume
6. **Press the canister 1 time and inhale at the same time**
7. **Inhale slowly and deeply**
8. Inhale continuously until achieving a full breath before taking the inhaler out of your mouth
9. Hold breath for at least 10 seconds
10. Wait about 1 minute before taking a 2nd dose (if needed)

Turbuhaler

1. **Remove cap**
2. **Keep inhaler in upright position**
3. **Turn red grip anti-clockwise then turn back until it "click"**
4. **Breath out gently away from the inhaler to residual volume**
5. Place the mouthpiece between the lips and teeth to seal the mouthpiece
6. **Inhale forcefully and deeply**
7. Inhale continuously until achieving a full breath before taking the inhaler out of your mouth
8. Breath out normally away from the inhaler
9. Wait about 1 minute before taking a 2nd dose (if needed)

Accuhaler

1. **Open cover**
2. **Push the lever until it "click"**
3. **Breath out gently away from the inhaler to residual volume**
4. Place the mouthpiece between the lips and teeth to seal the mouthpiece
5. **Inhale forcefully and deeply**
6. Inhale continuously until achieving a full breath before taking the inhaler out of your mouth
7. Hold your breath for at least 10 seconds
8. Breath out normally away from the inhaler

Handihaler/Breezhaler

1. **Open cover**
2. Open the mouthpiece
3. **Insert the capsule into the chamber**
4. Close the mouthpiece
5. **Pierce the capsule by fully pressing the button 1 time and then release**
6. **Breath out gently away from the inhaler to residual volume**
7. Place the mouthpiece between the lips and teeth to seal the mouthpiece
8. **Inhale deeply with capsule vibrating sound**
9. Inhale continuously until achieving a full breath before taking the inhaler out of your mouth
10. Hold your breath for at least 10 seconds
11. Breath out normally away from the inhaler
12. Inhale twice from the same capsule (repeat step 6-11 a second time)

Modified from previous study [18, 19]

Critical steps are shown in bold
