# Supplementary material for: Cognitive impairment according to Montreal Cognitive Assessment independently predicts the ability of chronic obstructive pulmonary disease patients to maintain proper inhaler technique
Source: BMC Pulm Med. 2023 Apr 26;23:144. doi: 10.1186/s12890-023-02448-x (PMC10131352; doi:10.1186/s12890-023-02448-x)
Supplement: Supplementary file 2 — Additional file 2: Supplementary Fig. 1. Receiver operating characteristic curve analysis of the Montreal Cognitive Assessment scores in patients with and without incorrect technique at 1 month after training. The optimal cut-off value was 16. [file 12890_2023_2448_MOESM2_ESM.docx]

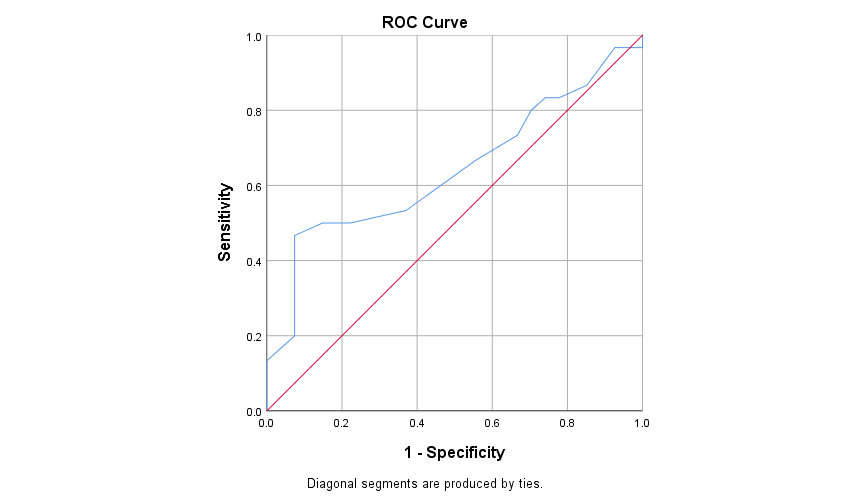


AUC = 0.643

95% CI: 0.497 − 0.788

**Supplementary Fig. 1.** Receiver operating characteristic curve analysis of the Montreal Cognitive Assessment scores in patients with and without incorrect technique at 1 month after training. The optimal cut-off value was 16.
